# Statistical inference in mechanistic models: time warping for improved gradient matching

**DOI:** 10.1007/s00180-017-0753-z

**Published:** 2017-08-09

**Authors:** Mu Niu, Benn Macdonald, Simon Rogers, Maurizio Filippone, Dirk Husmeier

**Affiliations:** 1 School of Mathematics and Statistics, University of Glasgow, Glasgow, UK; 2 Department of Computer Science, University of Glasgow, Glasgow, UK; 3 Data Science Department, Eurecom, Biot, France

**Keywords:** Differential equations, Reproducing kernel Hilbert space, Dynamical systems, Objective function

## Abstract

Inference in mechanistic models of non-linear differential equations is a challenging problem in current computational statistics. Due to the high computational costs of numerically solving the differential equations in every step of an iterative parameter adaptation scheme, approximate methods based on gradient matching have become popular. However, these methods critically depend on the smoothing scheme for function interpolation. The present article adapts an idea from manifold learning and demonstrates that a time warping approach aiming to homogenize intrinsic length scales can lead to a significant improvement in parameter estimation accuracy. We demonstrate the effectiveness of this scheme on noisy data from two dynamical systems with periodic limit cycle, a biopathway, and an application from soft-tissue mechanics. Our study also provides a comparative evaluation on a wide range of signal-to-noise ratios.

## Introduction

The scientific landscape is changing, with an increasing number of traditionally qualitative disciplines becoming quantitative and adopting mathematical modelling techniques. This change is most dramatically witnessed in the life sciences (Cohen 2004). One of the most widely used modelling paradigms is based on coupled ordinary or partial differential equations (DEs). These equations are typically non-linear, so that a closed-form solution is intractable and numerical solutions are needed. This usually does not pose any restrictions on the forward problem: given the parameters, generate data from the model. However, it does provide challenges for the backward problem: given the data, infer the parameters.

The simplest approach to parameter inference for DEs is to compare the solution of the equations, for some given parameter set, to noisy observations of the signal based on some appropriate noise model. Parameter estimation can then be carried out by minimizing the discrepancy between the predicted solution of the DEs and the data. Robinson (2004) contains an introduction for obtaining explicit solutions of differential equations and amongst many other topics, Robinson discusses the use of Euler’s method and the Runge–Kutta scheme as methods for obtaining solutions numerically. Inference could be carried out on a system of DEs by using either of these two methods (with a reasonably small step-size) to numerically solve the equations and use least squares estimation to infer the parameters that best describe the data signal. Xue et al. (2010) discuss the influence of the numerical approximation to the DEs (employing the 4-stage Runge–Kutta algorithm in their studies). They argue that previous studies took the numerical solution as being the ground truth and only considered the measurement error when estimating the parameters. The authors show that when the maximum step size of a *p*-order numerical algorithm goes to zero at a rate faster than $\displaystyle {n^{-1/p^4}}$, where *n* is the sample size, the numerical error is negligible in comparison to the measurement error. This provides some guidance in selecting the step-size when numerically solving DEs.

A different integration-based approach, which aims at avoiding explicitly solving the DEs, is to first smooth the data with a chosen interpolation method. This interpolant acts as a proxy for the solution of the DEs and the parameters can then be inferred with non-linear least squares. It is demonstrated in Xue et al. (2010) that a sieve estimator (a sequence of finite-dimensional models of increasing complexity) is asymptotically normal and has the same asymptotic covariance as when the true solution is known if the parameters are constant over time. A typical example of sieve regression is a spline (Hansen 2014). Dattner and Klaassen (2015) look at DEs where the systems are linear in the parameters. Taking advantage of the linearity in the model, the authors are able to develop a two-step estimation approach that does not require repeated integration of the system. By reformulating the minimization function in terms of integrals instead of derivatives, the authors obtain closed form estimates of the parameters of the system. These estimates are shown to be consistent estimators. Dattner and Klaassen consider two types of interpolation schemes—a local polynomial estimator and a step function estimator (which is obtained by averaging repeated measurements). The method using a local polynomial estimator was shown to outperform the two-step gradient matching approach of Liang and Wu (2008), whilst it was unable to outperform the gradient matching method of Ramsay et al. (2007). The accuracy of Daatner and Klaassen’s method using a step function estimator did not change much even when the number of repeated measures was quite small. Bayesian smooth-and-match is a related method, that avoids explicitly solving the DEs and instead indirectly solves the system by numerically integrating the interpolated signals. Ranciati et al. (2016) employ this approach, smoothing the data with penalized splines, and use ridge regression to infer the parameters of the DEs. Again, this approach focuses on systems that are linear in the parameters. In order to achieve a fully probabilistic generative model, the authors take a similar approach to Barber and Wang (2014) and as a consequence the vector of observations appears twice in the graphical model. The upshot of this is that the method is unable to deal with partially observed systems and the two observation vectors are coupled by a common nuisance (variance) parameter. Ranciati et al. (2016) demonstrate that the method is fast, with a built-in quantification of uncertainty about the DE solution. The results obtained, for a fully observed system that is linear in the parameters, are accurate and robust to dataset size and noise level.

In recent years, approximate methods based on gradient matching have been proposed. Here, the idea is to avoid the computationally expensive numerical solution of the DEs with an indirect approach, based on the following procedure: estimate the derivatives directly from the noisy data via some smoothing approach, quantify the discrepancy between these estimates and the predictions from the differential equations, and finally infer the model parameters based on this discrepancy. Methods can differ by the choice of interpolation scheme and the chosen metric for penalizing the difference between gradients. Wu et al. (2014) propose a five-step approach for inference in sparse additive ordinary differential equations (SA-ODE). The SA-ODE model is denoted as

$$\begin{aligned} {\dot{x}}_s= \chi _{s} + \sum _{i=1}^{N} f_{si}(x_{i}(t)) \end{aligned}$$

and it is assumed that the number of significant non-linear effects, $f_{si}(\cdot )$, is small for each of the *N* variables even though the total number of variables in the network may be large. At step one, the data is smoothed using penalized splines. At step two, the state variables and derivatives are substituted into the aforementioned SA-ODE model, producing a pseudo-sparse additive model (PSA). A truncated series expansion with B-spline bases is used to approximate the additive components of the PSA model. The number of basis functions is chosen as large as possible with the intention to correct for this at the fifth step. At step three, the group LASSO is used to identify significant functions in the model. The penalty parameter at this step is estimated using BIC. The group LASSO penalty treats the coefficients from each group equally, which is typically suboptimal. Hence, at step four, an adaptive group LASSO is applied to allow different levels of shrinkage to exist for different coefficients. Finally, at step five, a regular/adaptive LASSO is applied to account for the under-smoothing from step two (due to selecting more bases than are probably necessary). Wu et al demonstrate in their simulation studies that the method is able to obtain a high true positive rate, when the sample size is sufficiently large, and can more closely match the true underlying signal (noise free signal) than the method by Lu et al. (2011) which assumes a linear DE model and uses the smoothly clipped absolute deviation penalized likelihood method of Fan and Li (2001) for variable selection. A variety of other frameworks have also been developed in this context, including local linear and quadratic regression (Liang and Wu 2008), Gaussian processes (Calderhead et al. 2009; Dondelinger et al. 2013; Barber and Wang 2014; Macdonald et al. 2015), penalized smoothing and regression splines (Ramsay et al. 2007; Xun et al. 2013), and reproducing kernel Hilbert spaces (González et al. 2013, 2014).

A problem common to all of these approaches is the critical dependence of the inference scheme on the form of the interpolant. Small “wiggles”, which are hardly discernible at the level of the interpolant itself, can have dramatic effects at the level of the derivatives, which determine the parameter estimation. For noisy data, an adequate smoothing scheme is essential. However, any smoothing scheme is based on intrinsic length scales and these length scales may vary in time. Consider, for instance, estimating an oscillating signal with varying frequency using a Gaussian process (GP). If the length scale is tuned to the high-frequency domain, overfitting will typically result in the low frequency domain; if it is tuned to the low frequency domain, over-smoothing will affect the high frequency domain. In either case, the estimation of the derivatives will be poor, hampering DE parameter estimation.

The motivation for our work is given by the work of Calandra et al. (2016) in which the authors present examples where the smoothness assumptions upon which standard GPs are based are too restrictive. This limitation can be alleviated by mapping the data into a feature space. The authors integrate this map into what they call a manifold GP, and propose a joint inference scheme for learning both the transformation of the data and the GP regression from the feature space to the observed space.

The mapping proposed in Calandra et al. (2016) is, by the very nature of the inference scheme, a “black box”; for their practical work, the authors use a feedforward neural network. The modification we propose in the present article is to develop a map that explicitly targets changes in the length scales of oscillating signals. Periodic signals with varying lengths scales correspond to nonisotropic periodic limit cycles, and are characteristic of a large class of non-linear DEs (non-chaotic DEs without a stable fixed point).

**Fig. 1:**
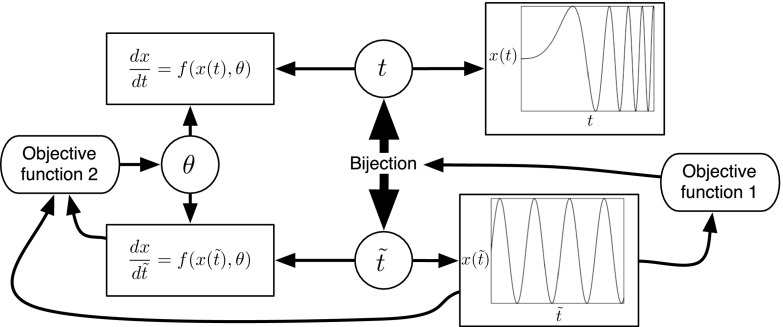
Graphical representation of the proposed method. A dynamical system, depending on the kinetic parameters $\theta$ (*top left*), has solutions subject to varying intrinsic length scales (*top right*). To improve inference, time *t* is warped into $\tilde{t}$ via a bijection (*centre*) with the objective to homogenize the intrinsic length scales (*bottom right*). This is achieved by minimizing an objective function that encourages functional invariance with respect to second-order differentiation (*far right*). The dynamical system in the warped domain can easily be obtained by application of the chain rule from standard calculus (*bottom left*). The kinetic parameters $\theta$ are then obtained by minimizing a second objective function based on gradient matching (*far left*). To avoid obfuscation, the figure does not specifically represent the distinction between the unknown true functions, *x*(*t*), and the interpolants used for their approximation, *g*(*t*) and $q(\tilde{t})$. A mathematically equivalent and more convenient way is to define the gradient matching in the original domain, after mapping the interpolants back into the original time domain. This has also not been shown, again to avoid obfuscation

The basic idea is that a regular sinusoid is easy to learn, whereas a quasi-periodic signal with varying frequencies is not. The objective, hence, is to find a warping of the time axis that counteracts the inhomogeneity in the period. This can easily be effected in principle. The characteristic feature of a regular sinusoid is the proportionality of the original function to its second derivative. Hence, we need to find a bijective transformation of time such that some metric quantifying the difference between the original function and a rescaled version of its second derivative is minimized in warped time. The procedure thus reduces to a double minimization problem, with respect to both the parameters of the map and the scaling parameter. An illustration is given in Fig. 1. The key difference to the work of Calandra et al. (2016) is that the map to be learned is explicit, with its own clearly defined objective function. A second difference is that our method is firmly integrated into the context of inference in differential equations, which provides the benchmark against which we assess performance.

In the present work, we implement the proposed warping scheme in the specific framework of reproducing kernel Hilbert space (RKHS) regression. We would like to emphasize, though, that this choice is rather arbitrary, and other regularized regression frameworks, like penalized splines or GPs, could also be chosen. The second point to notice is that although our framework has been motivated by oscillating functions, it turns out to be equally effective for non-periodic non-chaotic systems. We provide an example in the Results section (biopathway).

## Background

### Dynamical systems

Consider a dynamical system comprising *r* interacting states $x_s$, $1\le s\le r$, whose time evolution is governed by a set of coupled non-linear ordinary differential equations (DEs):

1 $$\begin{aligned} \dot{\varvec{x}}=\frac{d \varvec{x}}{d t}= \varvec{f}\left( \varvec{x}(t),\varvec{\theta } \right) , \end{aligned}$$

where $\varvec{x}=(x_1,\ldots ,x_r)$ is a time-dependent vector of state variables, and the parameters $\varvec{\theta }$ determine the kinetics of the interactions. Without loss of generality we will assume fixed initial conditions $\varvec{x}_0$. If these are unknown they can be integrated into the set of parameters $\varvec{\theta }$. We assume that we have time series of *n* noisy observations $\varvec{y}_s=(y_{s1},\ldots ,y_{sn})'$ of the states $\varvec{x}_s=(x_{s1},\ldots ,x_{sn})'$, subject to iid additive Gaussian noise $\varvec{\epsilon }_{k} \sim N(0, \sigma ^2\varvec{I})$:

2 $$\begin{aligned} \varvec{y}_{s} = \varvec{x}_{s} + \varvec{\epsilon }_{s} \end{aligned}$$

and the objective of inference is to learn $\varvec{\theta }$ from these noisy measurements.

### RKHS approach to inference in DEs

A Hilbert space ${\mathcal {H}}$ is a space of functions *g* defined over a set ${\mathbb {D}} \subset {\mathbb {R}}^m$. ${\mathcal {H}}$ is said to be a Reproducing Kernel Hilbert Space (RKHS) if and only if there exists a function $k(\cdot ,\cdot ): {\mathbb {D}} \times {\mathbb {D}} \rightarrow {\mathbb {R}}$ such that for all $t \in {\mathbb {D}}$ and all $g \in {\mathcal {H}}$ the inner product $<g( \cdot ), k(t,\cdot )>$ is equal to *g*(*t*) and the kernel function $k(t,\cdot )$ is in ${\mathcal {H}}$ (Aronszajn 1950). When working with an RKHS approach for function estimation, functions are expressed as a linear combination of kernel functions evaluated at the data points

3 $$\begin{aligned} x(t) = \sum _{i=1}^n b_i k(t, t_i) \end{aligned}$$

with $b_i \in {\mathbb {R}}$ and $t_i \in {\mathbb {D}}$. Many kernel functions are available including the squared exponential or Radial Basis Function (RBF) kernel, the spline kernel, and the multi-layer perceptron (MLP) kernel, to name just a few [see e.g. Bishop (2006), chapter 6].

In this framework, the unknown concentrations in Eq. (1) for the *s*th component of the dynamical system at time *t* (which implies $m=1$) can be modelled as

4 $$\begin{aligned} g_s(t;\varvec{b}_s) \; =\; \sum _{i=1}^{n}b_{si} k(t,t_i) \end{aligned}$$

with derivatives

5 $$\begin{aligned} {\dot{g}}_s(t;\varvec{b}_s)= & {} \sum _{i=1}^{n} b_{si} \frac{\partial k(t,t_i)}{\partial t} \; = \; \sum _{i=1}^{n} b_{si} {\dot{k}}(t,t_i) \end{aligned}$$

6 $$\begin{aligned} \ddot{g}_s(t;\varvec{b}_s)= & {} \sum _{i=1}^{n} b_{si} \frac{\partial ^2 k(t,t_i)}{\partial t^2} \; = \; \sum _{i=1}^{n} b_{si} \ddot{k}(t,t_i) \end{aligned}$$

where $\varvec{b}_s$ is the vector of kernel regression coefficients $b_{si}$. Following standard kernel ridge regression, smooth interpolants $g_s(t)$ are obtained by minimizing the following regularized loss function:

7 $$\begin{aligned} {\mathcal {L}}(\varvec{b}_s,\varvec{\varphi }_s;\lambda _s) = \sum _{i=1}^{n} \Big ( g_s( t_{i};\varvec{b}_s) - y_{s}(t_i) \Big )^2 + \lambda _s||\varvec{q}_s||^2 \end{aligned}$$

where $\varvec{\varphi }_s$ denotes the hyperparameters of the kernel function (e.g. the length scale of an RBF kernel),^1^ and the regularization term $||\varvec{q}_s||^2$ is the squared norm of ${\mathcal {H}}_s$:

8 $$\begin{aligned} ||\varvec{q}_s||^2 = \varvec{b}^T_s \varvec{K}_s \varvec{b}_s \end{aligned}$$

which contains a regularization parameter $\lambda _s\ge 0$. The minimization of ${\mathcal {L}}(\varvec{b}_s,\varvec{\varphi }_s;\lambda _s)$ with respect to $\varvec{b}_s$ for given $\varvec{\varphi }_s$ and $\lambda _s$ is a convex optimization problem with solution

9 $$\begin{aligned} \varvec{b}_s = (\varvec{K}_s+ \lambda _s \varvec{I} )^{-1} \varvec{y}_s \end{aligned}$$

Given $\lambda _s$, the kernel hyper-parameters $\varvec{\varphi }_s$ are optimized independently with a standard optimization routine, such as trust region or quasi-Newton. The regularization parameters $\lambda _s$ are estimated using tenfold cross validation.

Finally, the DE parameter $\varvec{\theta }$ can be estimated by minimizing the difference between $\dot{\varvec{g}}(t_i)$ and the gradient predicted from the DEs, $\varvec{f}( \varvec{g}(t_i),\varvec{\theta })$, using the following loss function:

10 $$\begin{aligned} L(\varvec{\theta }) \; = \; \sum _{s=1}^r \sum _{i=1}^{n} \Big [ {\dot{g}}_s( t_i) - f_s( \varvec{g}(t_i),\varvec{\theta }) \Big ]^2 \end{aligned}$$

where $f_s$ is the *s*th component of the function defined in (1). However, this approach critically depends on the expressive power of the linear combination of kernels to represent the solution of the DE system which in turn limits the flexibility of the representation of the solution of the DE system leading to a potential degradation of the performance in estimating DE parameters. For instance, in the case of an RBF kernel, rapid changes in the signals require a lengthscale parameter (which is included in $\varvec{\varphi }_s$) that is short enough to have sufficient flexibility to accommodate these changes. As a result, flat parts of the signal will be modelled with an unnaturally short lengthscale. This leads to overfitting, a poor estimation of the gradient and, consequently, a poor performance of gradient matching for DE parameter estimation (see Fig. 11 of the Appendix). In the next section, we describe a novel RKHS-based time warping approach to overcome this limitation.

## Methods

In order to overcome the difficulties imposed by variations in intrinsic functional length scales on smooth function interpolation, we introduce a two-layer approach. The objective of the first layer is to transform, for each of the variables *s* of the dynamical system, time *t* via a bijection $\tilde{t}=w_s(t)$ such that in warped time $\tilde{t}$, the unknown solutions $x_s$ of the dynamical system show less variation in their intrinsic length scales. More specifically, we target oscillating functions and aim to transform them into a regular sinusoid by exploiting the fact that a sinusoid is closed under second-order differentiation (subject to a rescaling). We define the transformation of time as

11 $$\begin{aligned} {\tilde{t}} = w_s(t,\varvec{b}^w,l^w)= & {} \sum _{j=1}^n \exp {\left( b_j^w\right) } \mathcal {S}(t - t_j, l^{w} ) \nonumber \\ \mathcal {S}(z,l^w)= & {} \frac{1}{1+ \exp (-l^w z)} \end{aligned}$$

where the strict monotonicity of $\mathcal {S}(.)$ and the non-negativity of $\exp (.)$ guarantee bijectivity. The number of basis functions *n* can, in principle, be treated as a model selection problem. In practice, we found that setting *n* to the actual number of observations gave satisfactory results (as reported in Sect. 5). In the original time domain, the *s*th variable of the dynamical system, $x_s(t)$, is approximated by the smooth interpolant $g_s(t)$. This function is now transformed, by virture of the bijection (11), into $q_s(\tilde{t})$, where

12 $$\begin{aligned} g_s(t) = q_s\circ w_s(t) = q_s({\tilde{t}}) \end{aligned}$$

and $w_s(t)$ is shorthand notation for the bijection defined in (11).

*Step 1: Initialization* We initialize the system with standard kernel ridge regression, i.e. by solving Eqs. (8–9). This gives us the smooth interpolants $g_s(t)$ in the original time domain *t*. We then initialize $\tilde{t}=t$ and $g_s(t) = q_s(\tilde{t})$, for each of the variables *s* of the dynamical system in turn.^2^

*Step 2: Time warping* The bijection between the original time domain $t \in [T_0,T_1]$ and the warped domain $\tilde{t} \in [\tilde{T}_0,\tilde{T}_1]$ is obtained by minimizing the objective function

13 $$\begin{aligned} L_w = \int \Big ( \ddot{ q}_s({\tilde{t}}) + [\lambda ^w]^2 q_s({\tilde{t}}) \Big )^2 d\tilde{t} + \lambda _t \left( \left( \tilde{T}_1 - T_1 \right) ^2 + \left( \tilde{T}_0 - T_0 \right) ^2 \right) \end{aligned}$$

The first term is minimized if $q_s(\tilde{t})$ is a regular oscillation (i.e. phase-shifted cosine or sinusoid) with angular frequency $\lambda ^w$. In practice, we usually have some prior knowledge about typical periods which can easily be incorporated by restricting the domain of $\lambda ^w$, e.g. by modelling it as the output of a rescaled sigmoidal function. The second term is a regularization term, weighted by a penalty parameter $\lambda _t > 0$, to discourage degenerate solutions. The practical choice of $\lambda _t$ is not critical as long as it is sufficiently large.^3^ The integral in (13) is analytically intractable and needs to be solved numerically, e.g. with the trapezoid or Simpson’s method. However, in practice, we only need the functional form of the bijection $w_s(.)$ at the observed time points $t_i, 1 \le i \le n$. This motivates the following simplification of the objective function (recall that $\tilde{t}_i=w_s(t_i)$):

14 $$\begin{aligned} L_w = \sum _{i=1}^n \Big ( \ddot{ q}_s({\tilde{t}}_i) + [\lambda ^w]^2 q_s({\tilde{t}}_i) \Big )^2 + \lambda _t \left( \left( \tilde{T}_1 - T_1 \right) ^2 + \left( \tilde{T}_0 - T_0 \right) ^2 \right) \end{aligned}$$

The parameters $\lambda ^w$, $l^w$ and $\varvec{b}^w$ are optimized by minimizing the loss function in Eq. (14), using gradient descent optimization.

The approximation of the integral in Eq. (13) by the finite sum in Eq. (14) is motivated by the fact that the gradients are only matched at the time points that are included in the sum. To control the smoothness of the warping function over the entire time domain, we found the following procedure useful. We apply standard kernel ridge regression with an MLP kernel [Bishop (2006), chapter 6] to the set of warped time points $\{ t_i,\tilde{t}_i) \}$ that have been obtained by minimizing Eq. (14). This gives us a new modified time warping function

15 $$\begin{aligned} {\hat{w}}_s(t) = \sum ^n_{j=1} b_{sj}^{{\hat{w}}} k_{mlp}(t,t_j) \end{aligned}$$

where $k_{mlp}(t,t_j)$ is a set of MLP kernel functions, and $b_{sj}^{{\hat{w}}}$ are the regression parameters, which are optimized by minimizing the following objective function:

16 $$\begin{aligned} {\mathcal {L}}\left( \varvec{b}_s^{{\hat{w}} },\lambda _s^{{\hat{w}}}\right) = \sum _{i=1}^{n} \Big ( {\hat{w}}_s( t_{i};\varvec{b}_s^{{\hat{w}}}) - \tilde{t}_i \Big )^2 + \lambda _s^{{\hat{w}}}||\varvec{{\hat{w}}}_s||^2 \end{aligned}$$

The roughness of the new warping function ${\hat{w}}_s(.)$ is controlled by the regularization parameter $\lambda _s^{{\hat{w}}}$, which is optimized by minimizing the loss function in Eq. (16) using leave-one-out crossvalidation. Examples of warping functions for four DE models used in simulation studies are shown in Appendix B.

*Step 3: Interpolation* The second layer deals with function interpolation. The original data points $y_s(t_i)$ are mapped to the warped time points, $y({\tilde{t}}_i)$. We then apply standard kernel ridge regression with an RBF kernel in the warped domain, resulting in a smooth interpolant $q_s({\tilde{t}})$, for each of the variables *s* in the dynamical system:

17 $$\begin{aligned} q_s\left( {\tilde{t}};\varvec{b^q}_s\right) \; =\; \sum _{j=1}^{n}b_{sj}^q k({\tilde{t}},{\tilde{t}}_j) \end{aligned}$$

Note that this interpolation problem is less susceptible to overfitting or oversmoothing, due to the fact that the initrinsic functional length scales (i.e. periods for an oscillating signal) have been homogenized by virtue of the time warping. Unwarping $q_s({\tilde{t}})$ back into the original time domain *t* is straightforward. Since $w_s(t)$ is bijective, we have $g_s(t)=q_s({\tilde{t}})$, and

18 $$\begin{aligned} \frac{d g_s(t)}{d t}=\frac{d q_s({\tilde{t}})}{d t} = \sum _{j=1}^{n}b_{sj}^q \frac{\partial k({\tilde{t}},{\tilde{t}}_j)}{\partial {\tilde{t}}} \frac{d {\tilde{t}}}{d t} = \sum _{j=1}^{n}b_{sj}^q \frac{\partial k({\tilde{t}},{\tilde{t}}_j)}{\partial {\tilde{t}}} w'_s(t) \end{aligned}$$

To illustrate the improvement afforded by warping, the gradient estimates with and without warping for four DE models used in simulation studies are presented in Appendix B.

*Step 4: Gradient matching* Finally, we estimate the DE parameters with standard gradient matching, i.e. by minimizing the following objective function^4^ with respect to $\varvec{\theta }$:

19 $$\begin{aligned} L(\varvec{\theta }) \; = \sum _{s=1}^r \sum _{i=1}^{n} \Big [ {\dot{g}}_s( t_i) - f_s( \varvec{g}(t_i),\varvec{\theta }) \Big ]^2 \; =\; \sum _{s=1}^r \sum _{i=1}^{n} \left[ \frac{ d q_s( {\tilde{t}}_i)}{d {\tilde{t}}_i} \frac{d {\tilde{t}}_i}{d t_i}- f_s( \varvec{q}({\tilde{t}}_i),\varvec{\theta }) \right] ^2 \end{aligned}$$

## Software

We have provided an implementation of the method to allow for reproducibility of our results. The code has been built in a modular, object oriented manner allowing flexibility and optimizing the opportunities for code re-use. The R package is available at http://dx.doi.org/10.5525/gla.researchdata.383.

## Simulations

The objective of our simulation study is to compare the performance of the novel two-level time warping method proposed in Sect. 3 with the standard RKHS gradient matching method summarized in Sect. 2.2. We refer to these methods as RKGW (W for warping) and RKG, respectively. Unless stated otherwise, we use an RBF kernel. For the comparative evaluation, we have generated time series from two well-known dynamical systems and a biopathway, and strain/stress data from a soft tissue mechanical model. To ensure a robust comparison, we have repeatedly and independently subjected these data to additive iid Gaussian noise, over a range of signal-to-noise ratios (SNR). The computational costs of the two approaches over the different DE models are shown in Table 8 of the Appendix.

*Lotka–Volterra* The Lotka–Volterra equations describe the dynamics of ecological systems with predator-prey interactions (Lotka 1920):

20 $$\begin{aligned} \begin{aligned} {\dot{x_1}}= \alpha \cdot x_1 - \beta \cdot x_1 \cdot x_2, \ {\dot{x_2}}= -\gamma \cdot x_2 + \delta \cdot x_1 \cdot x_2 \end{aligned} \end{aligned}$$

where the dot denotes a derivative with respect to time, $\alpha ,\beta ,\gamma ,\delta$ are four parameters to be inferred, and $x_1$ and $x_2$ are the states of the model, indicating the number of prey and predators respectively. We numerically solved the DEs for $\alpha =1$, $\beta =1$, $\gamma =4$, $\delta =1$ and initial conditions $x_1(0)=0.5$ and $x_2(0)=1$.

*FitzHugh–Nagumo* The FitzHugh–Nagumo system is a two-dimensional dynamical system used for modelling spike generation in axons (FitzHugh 1955). It has two state variables, $x_1$ and $x_2$, and three parameters: *a*, *b* and *c*.

21 $$\begin{aligned} \begin{aligned} {\dot{x_1}}= c\cdot \left( x_1 - x_1^3/3 + x_2 \right) , \ {\dot{x_2}}= -c^{-1} \left( x_1 -a + b \cdot x_2 \right) \end{aligned} \end{aligned}$$

*Biopathway* A model for the interactions of five protein isoforms, *S*, *dS*, *R*, *RS*,  *Rpp*, in a signal transduction pathway was studied by Vyshemirsky and Girolami (2008). The model describes interactions between the isoforms using both mass action and Michaelis–Menten kinetics:

22 $$\begin{aligned} \begin{aligned} {[}\dot{S}{]}&= - k_1 \cdot [S] - k_2 \cdot [S] \cdot [R] + k_3 \cdot [RS] \\ [\dot{dS}]&= k_1 \cdot \left[ S\right] \\ \dot{[R]}&= -k_2 \cdot [S] \cdot [R] + k_3 \cdot [RS] + \frac{k_5 \cdot [Rpp]}{k_6 + [Rpp]} \\ \dot{[RS]}&= k_2 \cdot [S] \cdot [R] - k_3 \cdot [RS]- k_4 \cdot [RS] \\ \dot{[Rpp]}&= k_4 \cdot [RS] - \frac{k_5 \cdot [Rpp]}{k_6 + [Rpp]} \end{aligned} \end{aligned}$$

The square brackets, $[\cdot ]$, denote concentrations, and the letters $k_{1:6}$ represent 6 kinetic parameters to be inferred. It turns out that $k_5$ and $k_6$ are only weakly identifiable, and we have thus assessed the accuracy of inference based on the ratio $\frac{k_5}{k_6}$. As ground truth, we took the kinetic parameters from Vyshemirsky and Girolami (2008).

*Soft tissue mechanics* We finally consider a soft-tissue mechanical model of the strain distribution in arteries that connect the human blood vessel network to the left ventricle of the heart. The arteries are modelled as a thick-walled non-linear elastic circular cylindrical tube. The deformation and the hyperelastic stress response of the arterial tissue material are described by the constitutive law proposed by Holzapfel and Ogden (2009), leading to

23 $$\begin{aligned} \frac{d \sigma }{dr}= & {} \frac{1}{r} \Big ( a \cdot e^{b\cdot (I_1(r)-3)} \cdot (\lambda _2(r)^2-\lambda _1(r)^2) \nonumber \\&+\, H\cdot a_f\cdot (I_4(r)-1) \cdot e^{b_f \cdot (I_4(r)-1)^2} \Big ) \end{aligned}$$

$$\begin{aligned} I_1= & {} \lambda _1^2 + \lambda _2^2 + \lambda _z^2, \ \ \lambda _1 = \frac{R}{t\cdot k \cdot \lambda _z} , \ \ \lambda _2 = \frac{k\cdot r}{R}, \\ R= & {} \sqrt{ (r^2-r_i^2)\cdot k \cdot \lambda _z + r_i^2 } , \\ I_4= & {} \lambda _2^2 \cdot \left( \cos ^2(\gamma ) + \lambda _z^2 \cdot \sin ^2(\gamma ) \right) , \\ \gamma= & {} \frac{2\pi }{3R_0-R_i} \cdot (R-R_i)-\frac{\pi }{3} \end{aligned}$$

Here, $\sigma$ is the strain and *r* is the radius of the tube. *H* is the indicator function, i.e. $H=1$ if $I_4>1$ and 0 otherwise. The constants $\lambda _z,R_i,R_0,k,r_i$ define known physiological properties that are predefined. The four patient-specific material parameters $a, b, a_f , b_f$ are of medical interest and need to be inferred from the experimental data; see Holzapfel et al. (2000).

**Fig. 2:**
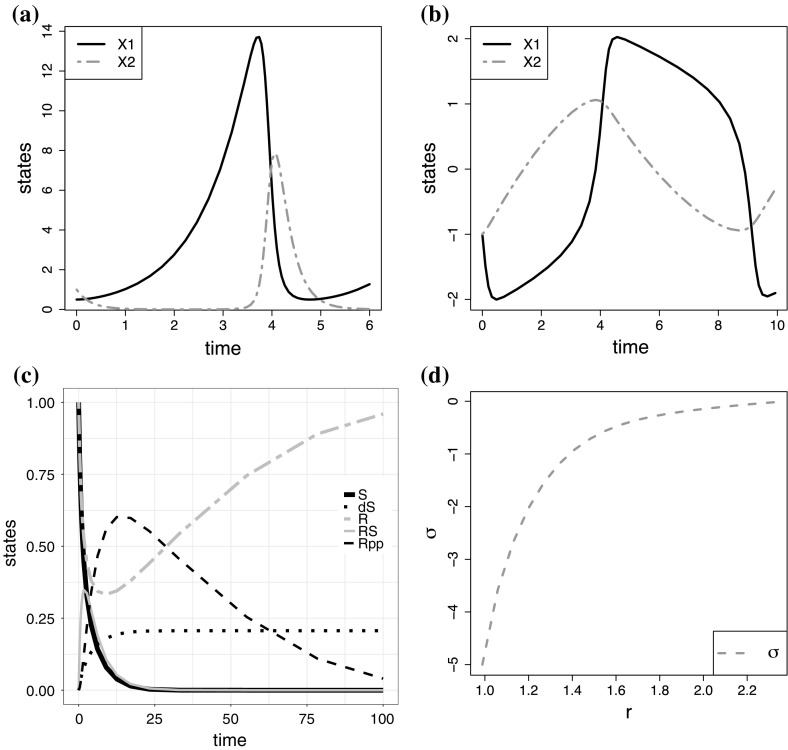
True solutions of the DE systems studied here. Note the inhomogeneity of the intrinsic length scales. **a** Lotka–Volterra, **b** FitzHugh–Nagumo, **c** Biopathway, **d** Soft tissue mechanics

For the Lotka-Volterra, FitzHugh-Nagumo and Biopathway model, the solutions of the three DE systems in time, as well as the time domains assumed in our study, are shown in Fig. 2a–c. The DEs were numerically integrated with a low-order Runge–Kutta method with automatic step-size adjustment, using the MATLAB function ODE23. We then uniformly downsampled the discrete time points by 50%, keeping every 2nd output from ODE23 (leading to $n=28, 37, 17$). We corrupted each data set with iid additive Gaussian noise with different standard deviations, corresponding to a range of signal-to-noise ratios (SNRs) between 10 and 40 db. For each SNR, we generated 50 independent noise instantiations.

For the Soft tissue mechanics model, the solution of the DE system, which shows the strain in arteries in response to changes of the blood vessel radius, is depicted in Fig. 2d. The DEs were numerically integrated and we chose $n=20$ equidistant radius values. The signal was corrupted with additive noise with SNR equal to 10 db, as assumed by our biological collaborators, and again we generated 50 independent data instantiations.

## Comparison with alternative state of the art methods

We have compared the proposed method with two related state-of-the-art methods from the recent literature: an alternative method also based on reproducing kernel Hilbert space regression (RKHS), proposed by González et al. (2013, 2014), and a method based on a graphical model representation with Gaussian processes, proposed by Barber and Wang (2014).

The alternative RKHS approach, henceforth refereed to as the GON method (after the first author, Gonzalez), is based on an explicit representation of the regularization operator $\varvec{K}_s$in Eq. (8) in terms of the differential operator (a product of the differential operator and its adjoint operator). Solutions of the homogeneous DE system are eigenfunctions, the so-called Greens functions, of this operator. In practice, a closed-form expression of the Greens functions is rarely available, and the differential operator has to be approximated by a finite difference operator. Additionally, the theory does not include non-homogeneous DEs with a non-linear function *f*(.) in Eq. (1). To make the method applicable to the general case, the authors linearize the system by replacing the state variables $\mathbf{x}(t)$ in the non-linear part of *f*(.) in Eq. (1) by fixed surrogates, obtained from, for example, a splines-based non-linear interpolation applied to the raw data.

The Gaussian process based approach, referred to by the authors as GPode, is based on a similar concept. Drawing on the analytical tractability of Gaussian processes, the state variables $\mathbf{x}(t)$ are first integrated out in closed form, to obtain the conditional probability of a noisy observation given the time derivatives of the state variables, ${{\dot{\mathbf{x}}}}(t)$, which can be directly linked to the explicit form of the DEs via Eq. (1). The graphical model is then conditioned on surrogates of the state variables $\mathbf{x}(t)$, which enter the DEs via Eq. (1).

## Results

**Fig. 3:**
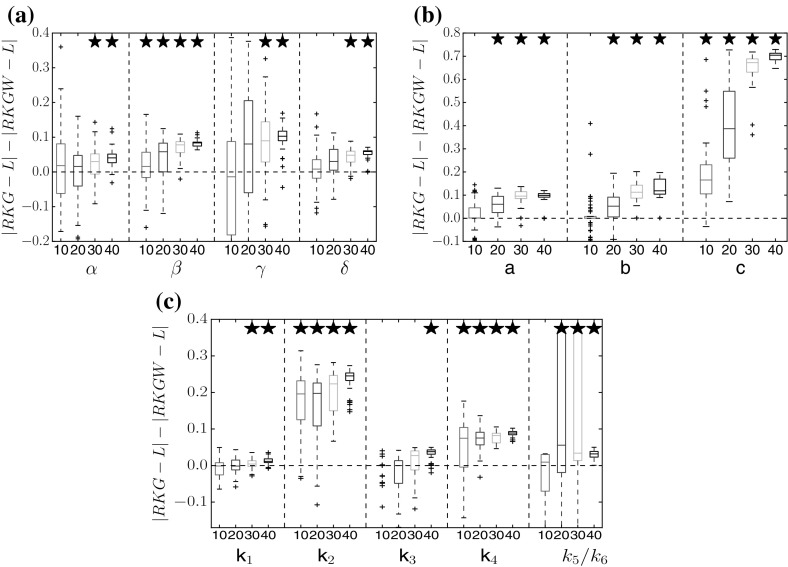
Method comparison in parameter space. Each box plot represents the distribution (from 50 independent noise instantiations) of differences between the absolute error of parameter estimates from the standard method (RKG: Sect. 2.2, no warping), and the absolute error of estimates from the proposed method (RKGW: Sect. 3, with time warping). Positive values (above the *dashed horizontal line*) indicate that time warping improves performance. The *horizontal axis* shows different signal-to-noise ratios for each DE parameter. *Asterisks above a box* indicate where the performance improvement is significant (based on a paired Wilcoxon test with 5% significance level). *Vertical axis*: RKGW is the estimate obtained with the proposed warping method (Sect. 3), RKG is the estimate obtained with the standard method without warping (Sect. 2.2), and L is the true value. Parameter distributions and *p*-values are provided in Tables 1, 2, 3 of Appendix A. **a** Lotka–Volterra, **b** FitzHugh–Nagumo, **c** Biopathway

**Fig. 4:**
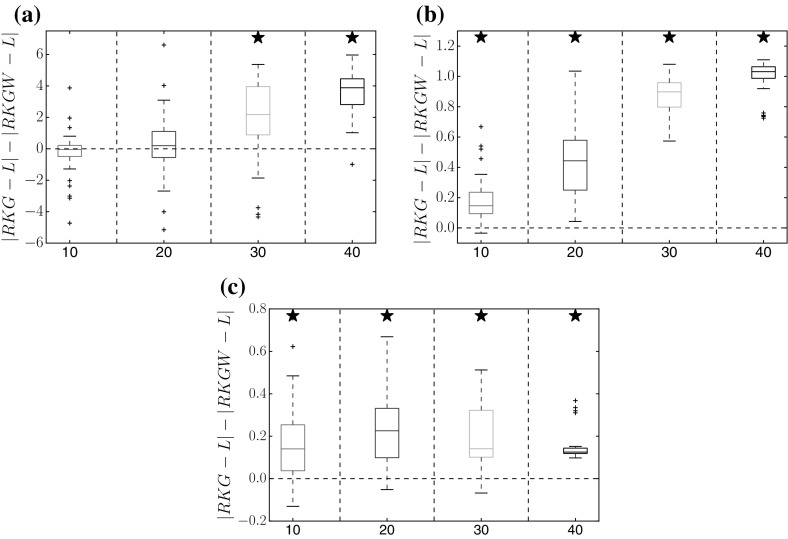
Method comparison in function space. Similar boxplot representation as in Fig. 3, but showing the distribution of the differences between the absolute errors of the function estimates; these function estimates are obtained by inserting the estimated parameters back into the DEs. Positive values indicate that the proposed method (warping) outperforms the standard method (no warping). *Asterisks* indicate that the improvement is significant (paired Wilcoxon test). Tables with *p*-values are available from Tables 1, 2, 3 of Appendix A. **a** Lotka–Volterra, **b** FitzHugh–Nagumo, **c** Biopathway

Figures 3 and 4 show a performance comparison between the proposed method (RKGW, Sect. 3) and the standard method without time warping (RKG, Sect. 2.2). Figure 3 shows a distribution of the difference of the absolute estimation error in parameter space; Fig. 4 shows a distribution of the absolute estimation error in function space, which is obtained by reinserting the inferred parameters into the DEs, numerically solving them, and then computing the rms difference between the solution and the true function. For all three benchmark systems, the proposed time warping method achieves a consistent improvement over the standard method for high SNRs (30 and 40 db). For low SNRs (10 and 20  db), the proposed method is significantly better in several instances, and never worse than the standard approach. We have carried out a series of paired Wilcoxon tests to formally test the null hypothesis of equal performance, with the *p*-values shown in Tables 1, 2, 3, 4, 5, 6, 7 of Appendix A. They confirm that the observed trends are statistically significant.

**Fig. 5:**
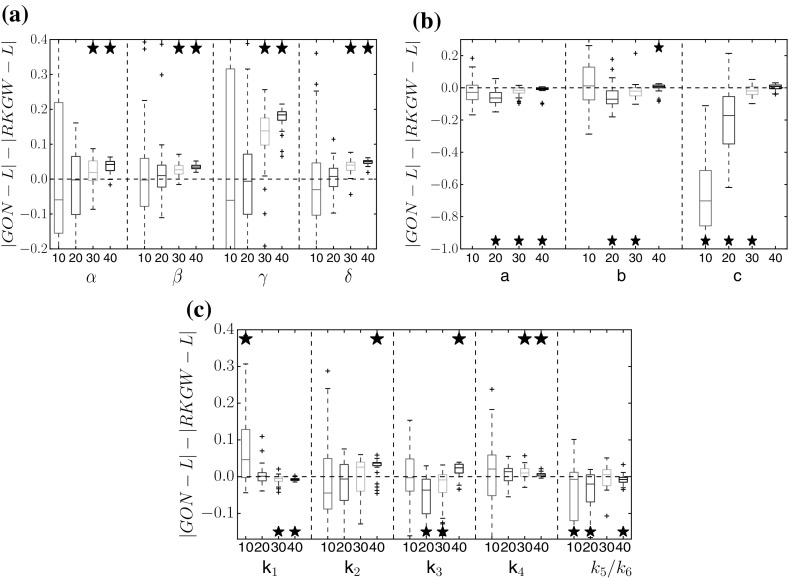
Comparison of RKGW and GON in parameter space. The box plots correspond to those in Fig. 3, but show a comparison between the proposed RKGW method and GON (González et al. 2013, 2014). *Asterisks above a box* indicate that the performance improvement with RKGW is significant (based on a paired Wilcoxon test). *Asterisks below a box* indicate that GON significantly outperforms RKGW. For further details, see the caption of Fig. 3. Tables with *p*-values are available from Tables 4, 5, 6 of Appendix A. **a** Lotka–Volterra, **b** FitzHugh–Nagumo, **c** Biopathway

**Fig. 6:**
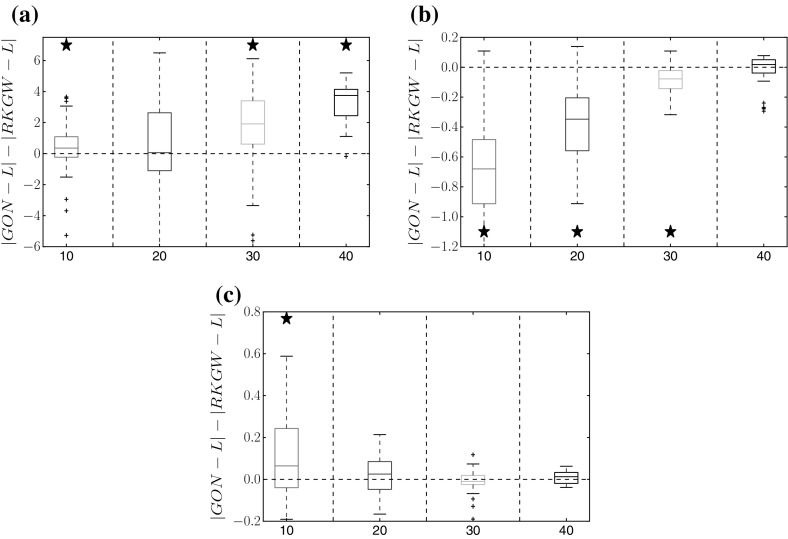
Comparison of RKGW and GON in function space. Similar boxplot representation as in Fig. 4, but showing a comparison between the proposed RKGW method and GON (González et al. 2013, 2014). *Asterisks above a box* indicate that the performance improvement with RKGW is significant (based on a paired Wilcoxon test). *Asterisks below a box* indicate that GON significantly outperforms RKGW. For further details, see the caption of Fig. 4. Tables with *p*-values are available from Tables 4, 5, 6 of Appendix A. **a** Lotka–Volterra, **b** FitzHugh–Nagumo, **c** Biopathway

Figures 5 and 6 show the corresponding comparisons with the GON method (González et al. 2013, 2014). For the Lotka–Volterra data, the proposed RKGW model is significantly better, For the FitzHugh–Nagumo data, the GON method is significantly better. For the biopathway data, both methods appear to be on a par, with sometimes GON, and sometimes RKGW performing significantly better.

**Fig. 7:**
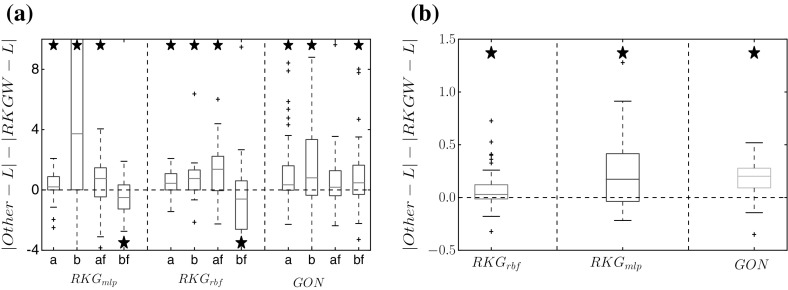
Comparison between RKGW and alternative methods for the soft-tissue mechanical model. Bias difference (i.e. difference of absolute differences from the true value, *L*) in parameter space (for $a,b,a_f,b_f$, see Eq. 23) (**a**), and in function space (**b**). In both cases, we compare the proposed warping method (RKGW) with three alternative methods: RKG without gradient matching, using an RBF kernel ($RKG_{rbf}$) and an MLP kernel ($RKG_{mlp}$), and the GON method. *Asterisks above the boxplot* indicate that the improvement obtained with the proposed method is significant (paired Wilcoxon test). For *asterisks below the boxplot*, the alternative method is significantly better. A table with *p*-values is available from Table 7 of Appendix A. **a** Parameter error, **b** functional error

Figure 7 compares the proposed RKGW method with two versions of the standard RKG method (with two different kernels: RBF and MLP) and with the GON method on the soft tissue mechanical data from Eq. (23), both in parameter space (panel a) and in function space (panel b). Here, RKGW consistently outperforms all other methods in function space, whilst in parameter space, it achieves a significant improvement in 9 out of 12 cases.

The comparison with GPode (Barber and Wang 2014) has been relegated to Appendix A. A naive application of this method, starting from a vague prior and no knowledge of the noise variance, consistently led to singularities with negative infinite log likelihoods, presumably due to the approximations inherent in GPode (integrating out the state variables and then reinserting them via surrogate variables; see Sect. 6). To get GPode to work, we had to use additional prior information (noise variance assumed to be known, informative parameter priors and informative parameter initialization). Still, we found that RKGW outperformed GPode on the Lotka–Volterra data, while for the other data, both methods were on a par (see Figs. 13, 14, 15, 16 in Appendix C.). Note that RKGW achieved this performance without the inclusion of additional prior information.

## Discussion

Inference in complex systems described by coupled differential equations (DEs) using gradient matching is challenging when the intrinsic length scales of functional change vary in the abscissa (time for dynamical systems, radius for the soft tissue mechanical model). In this article, we have proposed a time warping scheme to homogenize these length scales, based on an objective function that encourages functional invariance with respect to second-order differentiation. Applications to noisy data from three dynamical systems (Lotka–Volterra, FitzHugh–Nagumo, biopathway) have demonstrated consistent improvement over no warping for higher SNRs (30 and 40 db). For lower SNRs (10 and 20 db) the improvement was significantly improved in several cases, and never worse than for the standard scheme. For a soft tissue mechanical model with SNR $=$ 10 db, the proposed method significantly outperformed all other methods in function space, and for 3 out of 4 of the parameters.

We have carried out a comprehensive comparison with two alternative state-of-the-art methods from the recent literature: GON (González et al. 2013, 2014) and GPode (Barber and Wang 2014). At the face of it, all methods appear on a par. However, GPode showed considerable stability problems (see Appendix) and only achieved the presented level of performance when including a substantial amount of prior information, which the proposed RKGW method does not need (and did not include). GON outperformed the proposed method on the FitzHugh–Nagumo system. As seen from Eq. (21), this system of DEs has only a single non-linear term, so that the linearization approximation inherent in GON (as discussed in Sect.  6) appears to be less critical. For the Lotka–Volterra system (Eq. 20) and the soft tissue mechanical model (Eq. 23), on the other hands, where the DEs include more substantial non-linear contributions and the linearization assumption inherent in GON is more critical, the RKGW warping method that we have proposed achieves a significant performance improvement.

**Fig. 8:**
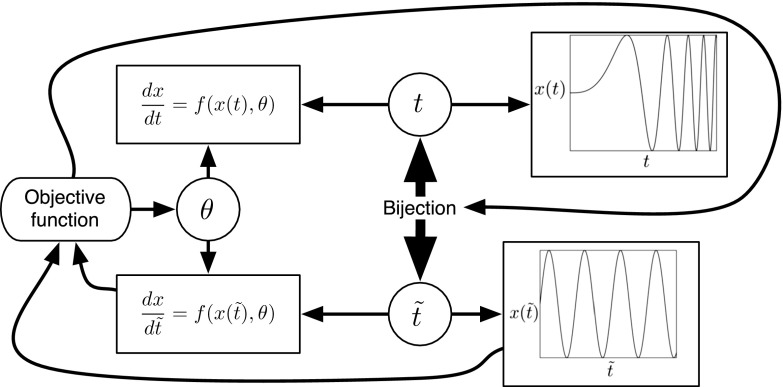
Learning time warping with a single objective function. The figure shows a modification of the method proposed in our paper to pursue the time warping more in line with the method proposed by Calandra et al. (2016). Rather than using a separate objective function that specifically aims to homogenize the smoothness characteristics of the underlying processes, as in Fig. 1, a time warping is learned that aims to optimize the same objective function as used for learning the DE parameters

The motivation for the proposed scheme comes from the idea of manifold Gaussian processes (Calandra et al. 2016). The objective of the paper by Calandra et al. (2016) is to alleviate the problem of learning complex functions by transforming the data into a feature space such that the regression task becomes easier in the new latent representation. This latent feature space is learned along with the actual function in a supervised manner. Typical applications where the proposed approach achieves improved results are high-dimensional processes confined to low-dimensional manifolds, as their successful identification reduces the effect of the curse of dimensionality. The authors also demonstrate that their approach can learn time warpings that alleviate function regression. Common to many regression methods, like Gaussian processes and kernel ridge regression, are smoothness assumptions about the functions to be modelled. These assumptions are too restrictive if the smoothness characteristics change in time, leading to poor interpolants that do not match the true underlying functions. Warping the original time axis into a transformed space in which the smoothness characteristics are more uniform can then lead to improved regression results, as both Calandra et al. (2016) and we show in our papers. The essential difference between the two approaches is shown in Figs. 1 and 8. In Calandra et al. (2016), the model used for performing the time warping (e.g. a multilayer perceptron, as used by the authors) has to figure out the warping strategy on its own, as part of an overall supervised learning process. Note that time warping is only one of many applications of the authors’ method, along with manifold learning and the identification of low-dimensional subspaces for high-dimensional functions, as described above. Our method, on the other hand, is solely focussed on learning scalar functions in time, as part of the wider problem of parameter inference in systems of coupled differential equations. For that reason, we encapsulate the homogenization strategy—the strategy that renders the smoothness characteristics more homogeneous in time—in a separate objective function. While our approach lacks the universal nature of manifold learning, it is ideally suited for temporal regression, as the homogenization of smoothness characteristics is the very objective of learning and does not have to be figured out by the learning machine on its own. To paraphrase that: Since we are not interested in manifold learning in general, but in parameter estimation of differential equations, we use a transformation into a ‘feature space’ that is solely focussed on time warping. Due to this focussed nature, the training scheme can make use of additional ‘prior knowledge’ (i.e. the homogenization strategy), which is encapsulated in a separate objective function.

There is also a potential connection with the method proposed in Su et al. (2014). This paper deals with trajectories on Riemannian manifolds, and the problem that the authors discuss is the observation of trajectories at random times, which may bias the mean trajectory and artificially inflate the variance over a population. The authors show that this can be formulated as a time warping problem. Consider two trajectories $\alpha _1$ and $\alpha _2$, where the first trajectory is formulated as a function of time *t*, and the second trajectory is formulated as a function of warped time $\tilde{t}$, which is a smooth bijective function of the real time axis into itself. A standard approach for finding the optimal warping function $\tilde{t}(t)$, referred to as ‘registration’ in Su et al. (2014), is to minimize the following functional:

24 $$\begin{aligned} E(\tilde{t}) \; = \; \min _{\tilde{t}} \left\{ \int \Big [\alpha _1(t)-\alpha _2(\tilde{t}[t])\Big ]^2 dt \; + \; \lambda \mathcal {R}(\tilde{t}) \right\} \end{aligned}$$

where $\lambda \ge 0$ is a regularization parameter, and $\mathcal {R}$ is a regularization function. The Euclidean norm can be generalized to geodesic distances on arbitrary Riemannian manifolds. However, the authors point out that this functional is not symmetric with respect to label swapping $1\leftrightarrow 2$, and that the minimum value is not a proper metric. They address this problem by defining a new distance function based on a square-root velocity vector field, which turns out to be a proper metric.

Rather than quantify the similarities between two trajectories, we quantify the similarities between two derivative curves in this work: one estimated by interpolation directly from the data (call this $\alpha _1(t)$), the other predicted by the model, and hence dependent on the differential equation parameters ${\varvec{\theta }}$; call this $\alpha _2(t,{\varvec{\theta }})$. This gives, in modification of Eq. (24):

25 $$\begin{aligned} E(\tilde{t}) \; = \; \min _{\tilde{t}} \left\{ \int \Big [\alpha _1(\tilde{t}[t])-\alpha _2(\tilde{t}[t],{\varvec{\theta }})\Big ]^2 dt \; + \; \lambda \mathcal {R}(\tilde{t}) \right\} \end{aligned}$$

Note that the essential difference from Eq. (24) is the symmetrization as a consequence of the fact that time warping, $t \rightarrow \tilde{t}$, enters both functions $\alpha _1$ and $\alpha _2$ equally. This renders the method extension described above obsolete. It opens up a potential other problem discussed in Su et al. (2014), though: if two trajectories are subjected to the same time warping $\tilde{t}$, then the distance between them should be independent of $\tilde{t}$. Su et al. (2014) show that their proposed distance function based on the square-root velocity vector field achieves this objective. We note again the essential difference between the two problems. Su et al. (2014) compare actual trajectories from a population of similar individuals (e.g. flocks of birds). We, on the other hand, deal with a noisy interpolation problem, and Eq. (25) quantifies the discrepancy between the interpolant and the DE model. As we have shown in our paper, the difficulty of the noisy interpolation problem depends on the time warping, and a more reliable interpolant will be more consistent with the DE model. For that reason, it is natural and intuitive that our metric depends on the time warping.

Finally, as discussed in Section 5 of Su et al. (2014), it is natural to generalize the Euclidean metric to the geodesics of an arbitray Riemannian manifold e.g. in trajectories of images in video surveillance. However, this is less of an issue for low-dimensional functions in time. A closer investigation of this aspect could provide a topic for future research.

A natural continuation of our work would be a model extension along the lines of the hierarchical Bayesian modelling framework proposed in Section 3 of Xun et al. (2013), whereby the DEs shape the prior distribution over the parameters. This framework would naturally benefit from the homogenization of the intrinsic functional length scales achieved with the proposed scheme. Our investigations have provided a first proof-of-principle study. They also provide a quantification of the improvement in the accuracy of inference that can be achieved, over a wide range of signal-to-noise ratios.

## Figures and Tables

**Fig. 9:**
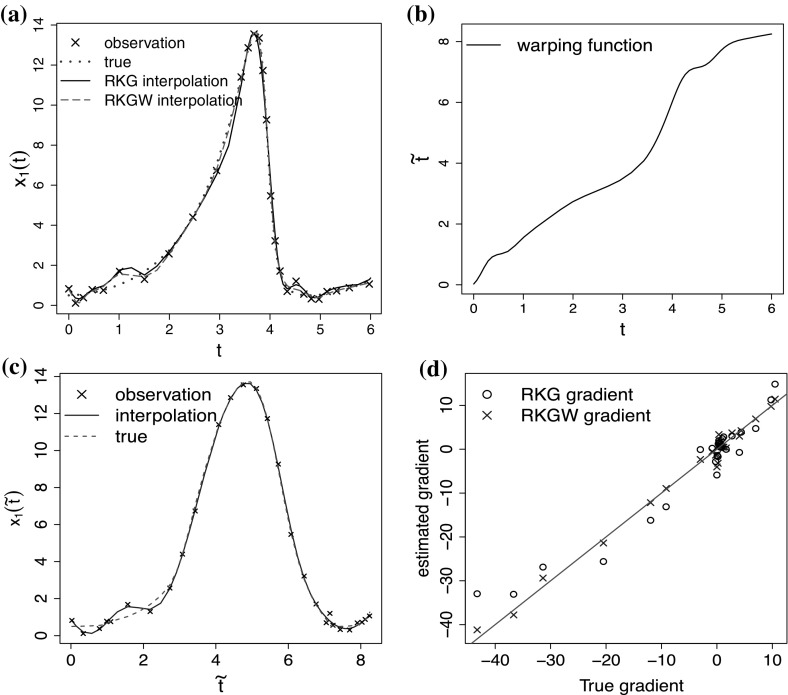
Warping example for Lotka-Volterra. The true signal (*dotted*), the 20 db SNR noisy data, the initial interpolation using the standard RBF kernel RKHS regression (RKG) (*solid*) and the interpolation using warping (RKGW) (*dashed*) are shown in (**a**). The warping function is shown in (**b**). The interpolation in the warped time domain using the RBF kernel (*blue solid*) and the warped true signal (*red dashed*) are shown in (**c**). The gradients estimated using RKG and RKGW are plotted against the true gradients in the scatterplot in (**d**). The rms error between the estimated and true gradients has been reduced from 3.37 for RKG to 1.45 for RKGW (color figure online)

**Fig. 10:**
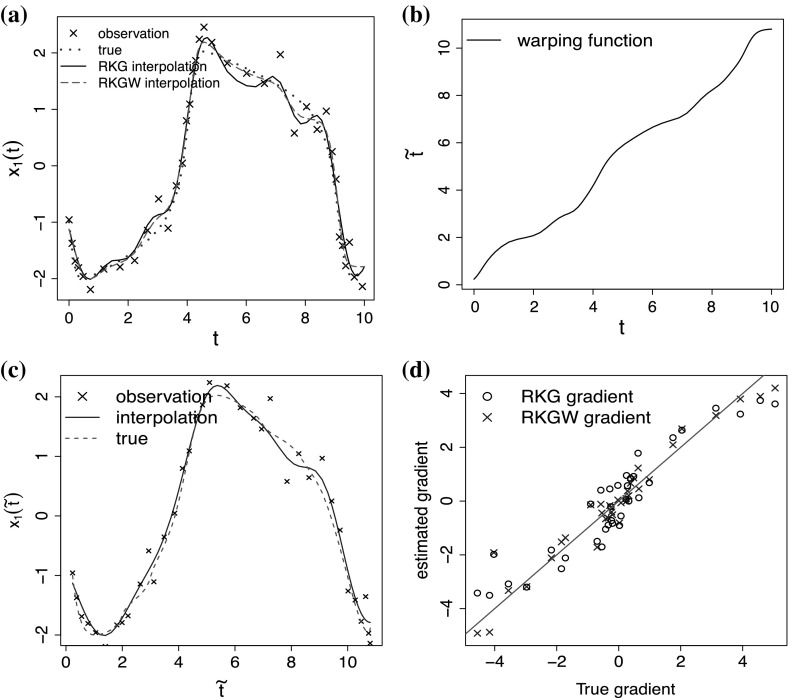
Warping example for FitzHugh-Nagumo. The true signal (*dotted*), the 10 db SNR noisy data, the initial interpolation using the standard RBF kernel RKHS regression (RKG) (*solid*) and the interpolation using warping (RKGW) (*dashed*) are shown in (**a**). The warping function is shown in (**b**). The interpolation in the warped time domain using the RBF kernel (*blue solid*) and the warped true signal (*red dashed*) are shown in (**c**). The gradients estimated using RKG and RKGW are plotted against the true gradients in the scatterplot in (**d**). The rms error between the estimated and true gradients has been reduced from 0.75 for RKG to 0.53 for RKGW (color figure online)

**Fig. 11:**
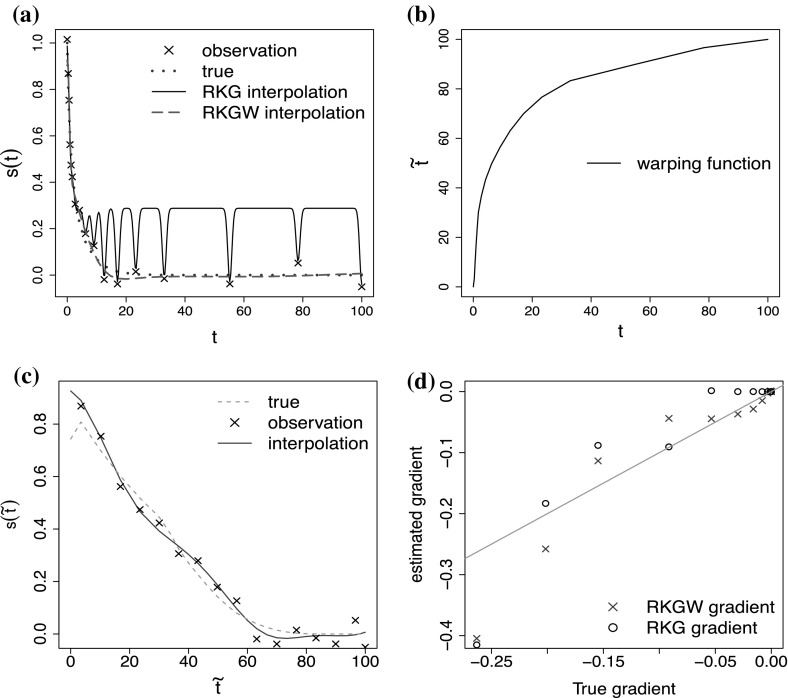
Warping example for the Biopathway model. The true signal (*dotted*), the 10 db SNR noisy data, the initial interpolation using the standard RBF kernel RKHS regression (RKG) (*solid*) and the interpolation using warping (RKGW) (*dashed*) are shown in (**a**). RBF regression is unable to cope with both the rapid drop and the saturated section of the curve. The warping function is shown in (**b**). The interpolation in the warped time domain using the RBF kernel (*blue solid*) and the warped true signal (*red dashed*) are shown in (**c**). The gradients estimated using RKG and RKGW are plotted against the true gradients in the scatterplot in (**d**). The rms error between the estimated and true gradients has been reduced from 4.8e−2 for RKG to 4.4e−2 for RKGW (color figure online)

**Fig. 12:**
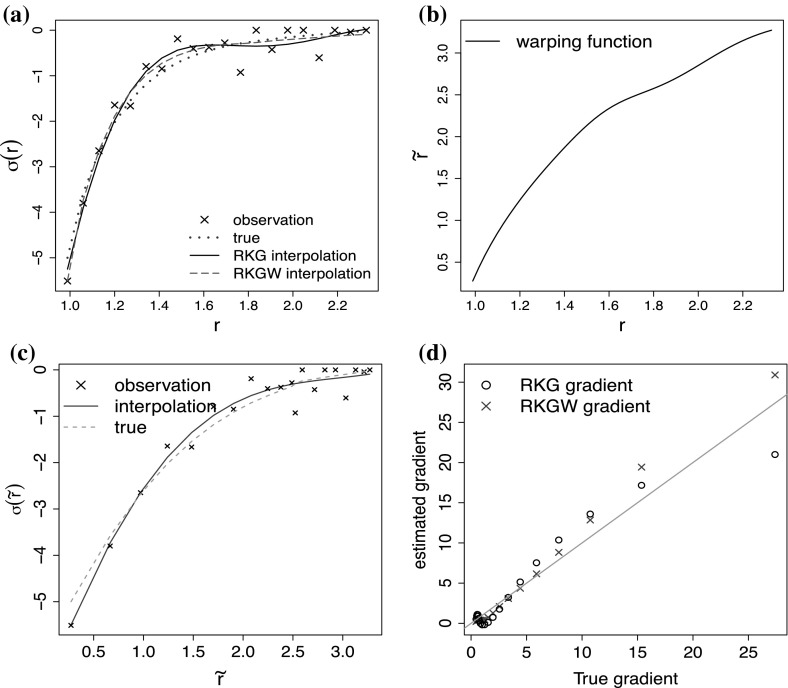
Warping example for the Soft tissue mechanics model. The true signal (*dotted*), the 10 db SNR noisy data, the initial interpolation using the standard RBF kernel RKHS regression (RKG) (*solid*) and the interpolation using warping (RKGW) (*dashed*) are shown in (**a**). The warping function is shown in (**b**). The interpolation in the warped time domain using the RBF kernel (*blue solid*) and the warped true signal (*red dashed*) are shown in (**c**). The gradients estimated using RKG and RKGW are plotted against the true gradients in the scatterplot in (**d**). The rms error between the estimated and true gradients has been reduced from 1.88 for RKG to 1.36 for RKGW (color figure online)

**Fig. 13:**
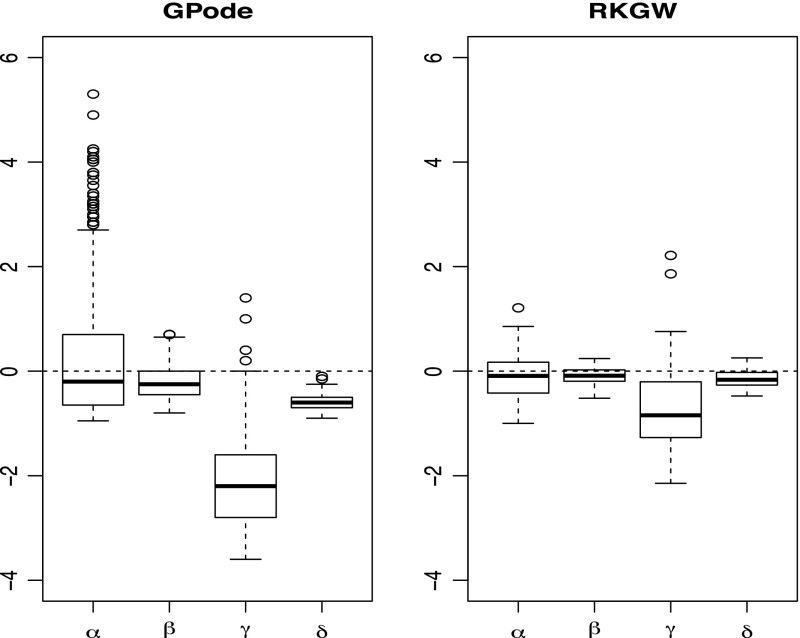
Warping versus GPode for the Lotka-Volterra model at 10 db SNR, values in parameter space. The *boxes* represent the distributions of the parameter differences (inferred value minus true value). *Left panel* GPode. *Right panel* the proposed warping method, RKGW. The *dashed horizontal line* indicates no difference from the true value

**Fig. 14:**
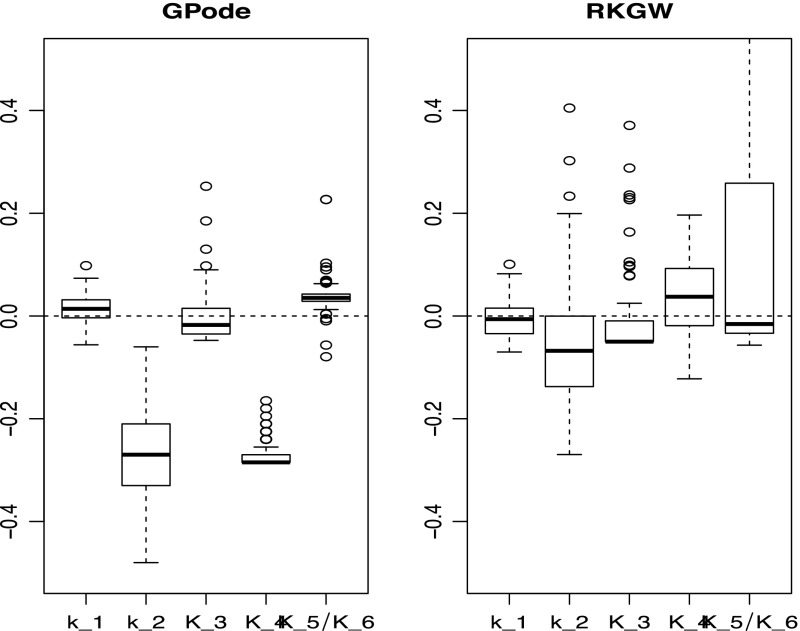
Warping versus GPode for the Biopathway model at 10 db SNR, values in parameter space. The *boxes* represent the distributions of the parameter differences (inferred value minus true value). *Left panel* GPode. *Right panel* the proposed warping method, RKGW. The *dashed horizontal line* indicates no difference from the true value

**Fig. 15:**
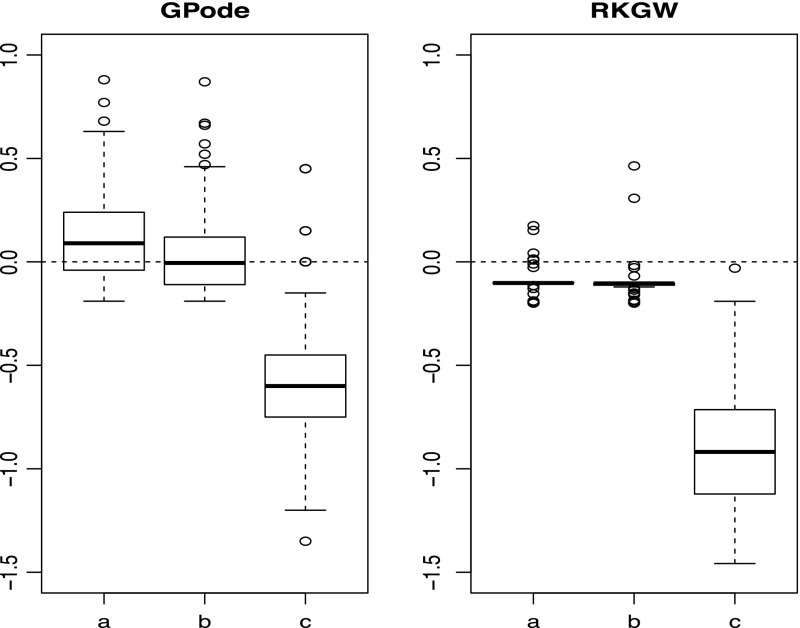
Warping versus GPode for the FithHugh–Nagumo model at 10 db SNR, values in parameter space. The *boxes* represent the distributions of the parameter differences (inferred value minus true value). *Left panel* GPode. *Right panel* the proposed warping method, RKGW. The *dashed horizontal line* indicates no difference from the true value

**Fig. 16:**
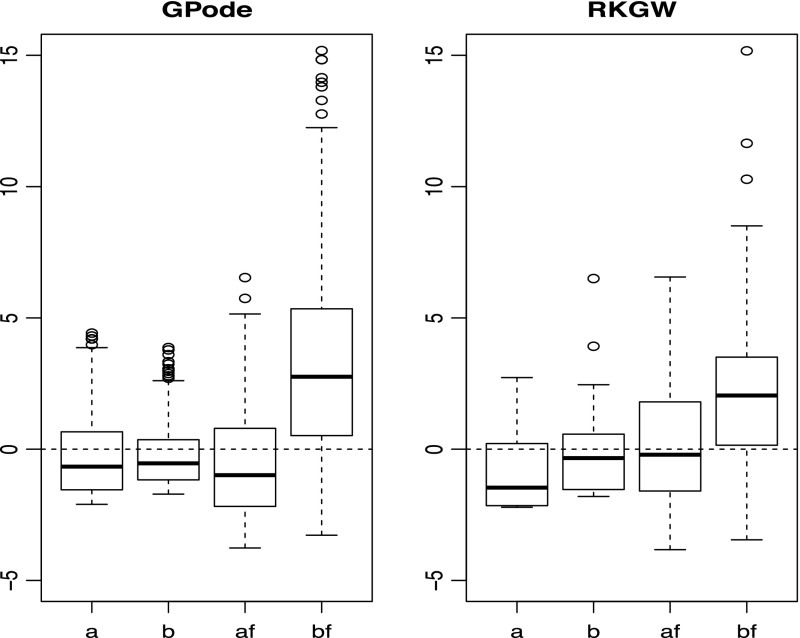
Warping versus GPode for the soft-tissue mechanics model at 10 db SNR, values in parameter space. The *boxes* represent the distributions of the parameter differences (inferred value minus true value). *Left panel* GPode. *Right panel* the proposed warping method, RKGW. The *dashed horizontal line* indicates no difference from the true value

**Fig. 17:**
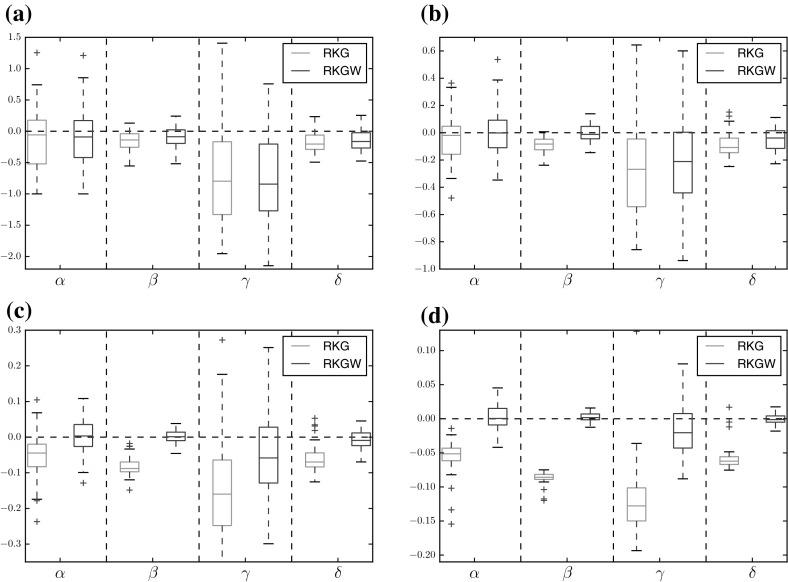
Warping versus no warping for the Lotka-Volterra model at all SNR values in parameter space. The figure shows the distributions of parameter estimates from 50 data instantiations. The *boxes* represent the distributions of the parameter differences (inferred value minus true value). *Red* is for no warping and *blue* is for warping. The *dashed horizontal line* indicates no difference from the true value. **a** SNR 10 db, **b** SNR 20 db, **c** SNR 30 db, **d** SNR 40 db (color figure online)

**Fig. 18:**
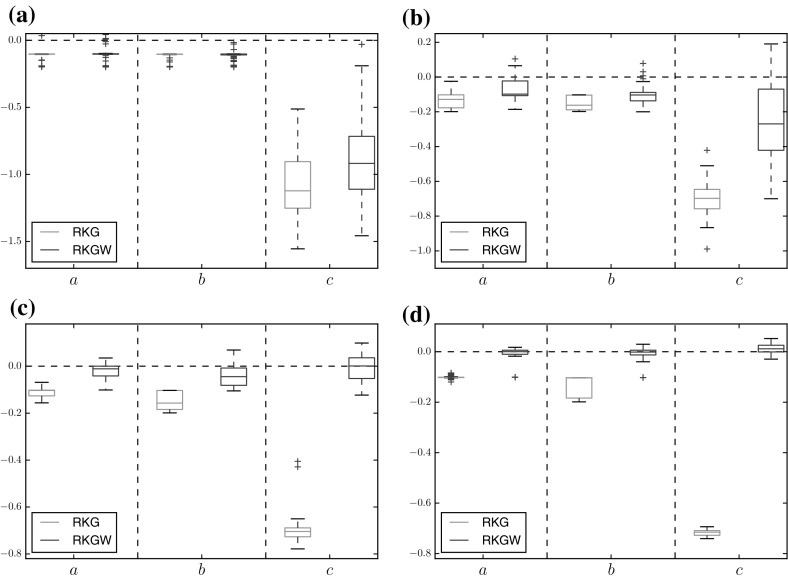
Warping versus no warping for the FitzHugh-Nagumo model at all SNR values in parameter space. The figure shows the distributions of parameter estimates from 50 data instantiations. The *boxes* represent the distributions of the parameter differences (inferred value minus true value). *Red* is for no warping and *blue* is for warping. The *dashed horizontal line* indicates no difference from the true value. **a** SNR 10 db, **b** SNR 20 db, **c** SNR 30 db, **d** SNR 40 db (color figure online)

**Fig. 19:**
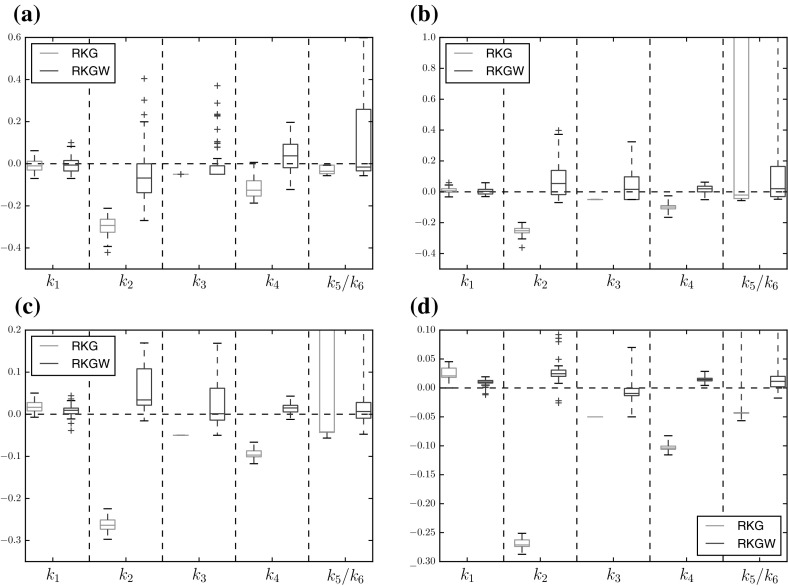
Warping versus no warping for the Biopathway model at all SNR values in parameter space. The figure shows the distributions of parameter estimates from 50 data instantiations. The *boxes* represent the distributions of the parameter differences (inferred value minus true value). *Red* is for no warping and *blue* is for warping. The *dashed horizontal line* indicates no difference from the true value. **a** SNR 10 db, **b** SNR 20 db, **c** SNR 30 db, **d** SNR 40 db (color figure online)

## A *p*-value tables

The following tables show the *p*-values for the comparisons of parameter estimates between the proposed warping method and an RKHS method without warping.

**Table 1:** Method evaluation on the Lotka–Volterra data

*SNR*	Method	par${\scriptscriptstyle 10^{-2}}$	fun	$\alpha$	$\beta$	$\gamma$	$\delta$
40 db	*RKGW*	0.9 (0.5)	1.19 (0.97)	**6e**−**15**	**4e**−**47**	**3e**−**25**	**3e**−**29**
	*RKG*	4.5 (0.7)	5.06 (0.54)				
30 db	*RKGW*	2.6 (2)	2.64 (2.37)	**2.8e**−**4**	**1e**−**22**	**5e**−**8**	**8-e15**
	*RKG*	6 (2.1)	5.06 (1.81)				
20 db	*RKGW*	8.6 (6.2)	5.86 (1.9)	0.92	**9e**−**6**	0.98	0.41
	*RKG*	11 (6.5)	5.87 (2.19)				
10 db	*RKGW*	29 (15)	6.98 (0.82)	0.86	**0.03**	0.41	0.28
	*RKG*	27 (16)	6.9 (0.85)				

Performance criteria are the root median square error in parameter space (par) and function space (fun; for the functions obtained by inserting the estimated parameters into the DEs). Values in brackets show the median absolute deviation (MAD). The true parameter values are: $\alpha =1$, $\beta =1$, $\gamma =4$, $\delta =1$; the corresponding true functions are shown in panel (a) of Fig. 2. A paired *t*-test was carried out to test the statistical significance of the absolute differences, $|RKGW-L|-|RKG-L|$, where *RKGW* is the estimate obtained with the proposed method, discussed in Sect. 3, *RKG* is the estimate obtained with the standard method, summarized in Sect. 2.2, and *L* is the true value. The corresponding *p*-values are listed; values shown in bold indicate a significant improvement obtained with the proposed method

**Table 2:** Method evaluation on the FitzHugh–Nagumo data

*SNR*	Method	par${\scriptscriptstyle 10^{-2}}$	fun	*a*	*b*	*c*
40 db	*RKGW*	0.6 (0.4)	0.06 (0.04)	**1e**−**25**	**8e**−**21**	**5e**−**76**
	*RKG*	25.5 (1.1)	1.09 (0.02)			
30 db	*RKGW*	2 (0.9)	0.17 (0.09)	**4e**−**20**	**2e**−**19**	**5e**−**50**
	*RKG*	25.2 (2)	1.08 (0.19)			
20 db	*RKGW*	7.7 (4.6)	0.61 (0.25)	**8e**−**10**	**5e**−**7**	**2e**−**22**
	*RKG*	25.5 (4.3)	1.07 (0.08)			
10 db	*RKGW*	23 (6.9)	1.28 (0.3)	0.77	0.32	**2e**−**12**
	*RKG*	31.3 (15.6)	1.47 (0.27)			

The true parameter values are: $a=0.2$, $b=0.2$, $c=3$; the corresponding true functions are shown in panel (b) of Fig. 2. For explanations, see the caption of Table 1

The *p*-values corresponding to the individual parameters are shown in the last three columns. Values shown in bold indicate a significant improvement achieved with the proposed method

**Table 3:** Method evaluation on the Biopathway data

*SNR*	Method	par${\scriptscriptstyle 10^{-2}}$	fun${\scriptscriptstyle 10^{-2}}$	$k_1$	$k_2$	$k_3$	$k_4$	$\frac{k_5}{k_6}$
40 db	*RKGW*	0.9 (0.1)	5.5 (0.7)	**9.4e**−**12**	**1.8e**−**46**	**5.1e**−**18**	**5.4e**−**56**	0.12
	*RKG*	8.4 (2.6)	18 (1.1)					
30 db	*RKGW*	1.3 (0.6)	6 (2.1)	**4.5e**−**3**	**2e**−**28**	0.4	**1.1e**−**35**	0.09
	*RKG*	8.7 (1.3)	18.9 (5.2)					
20 db	*RKGW*	3.5 (2.5)	8.9 (5.4)	0.94	**1.8e**−**16**	9e−3	**2.9e**−**22**	**1.5e**−**3**
	*RKG*	10.6 (4.2)	33.6 (21.7)					
10 db	*RKGW*	5.3 (3.1)	16.6 (6.3)	0.091	**4.4e**−**20**	1.2e−2	**6e**−**4**	0.32
	*RKG*	10.2 (2)	28.1 (13.5)					

The true parameter values are: $k_1=0.07$, $k_2=0.6$, $k_3=0.05$, $k_4=0.3$ and $\frac{k_5}{k_6}=0.057$; the corresponding true functions are shown in panel (c) of Fig. 2. For explanations, see the caption of Table 1

The *p*-values corresponding to the individual parameters are shown in the last five columns. Values shown in bold indicate a significant improvement achieved with the proposed method

**Table 4:** Comparison of RKGW and GON on the Lotka–Volterra data

*SNR*	Method	par${\scriptscriptstyle 10^{-2}}$	fun	$\alpha$	$\beta$	$\gamma$	$\delta$
40 db	*RKGW*	0.9 (0.5)	1.19 (0.97)	**1e**−**9**	**7e**−**10**	**7e**−**10**	**7e**−**10**
	*GON*	5.7 (0.5)	4.81 (0.54)				
30 db	*RKGW*	2.6 (2)	2.64 (2.37)	**5e**−**3**	**1e**−**10**	**2e**−**8**	**6-e12**
	*GON*	6.2 (2)	4.76 (1.71)				
20 db	*RKGW*	8.6 (6.2)	5.86 (1.9)	0.77	0.23	0.63	0.53
	*GON*	9 (4.3)	6.34 (1.28)				
10 db	*RKGW*	29 (15)	6.98 (0.82)	0.93	0.98	0.52	0.3
	*GON*	23 (13)	7.24 (0.73)				

GON is the estimate obtained with Gonzalez (2014), summarized in Sect. 6. For explanations, see the caption of Table 1

The *p*-values corresponding to the individual parameters are shown in the last four columns. Values shown in bold indicate a significant improvement achieved with the proposed method

**Table 5:** Comparison of RKGW and GON on the FitzHugh–Nagumo data

*SNR*	Method	par${\scriptscriptstyle 10^{-2}}$	fun	*a*	*b*	*c*
40 db	*RKGW*	0.6 (0.4)	0.06 (0.04)	**3e**−**7**	**5e**−**3**	0.15
	*GON*	0.4 (0.2)	0.07 (0.02)			
30 db	*RKGW*	2 (0.9)	0.17 (0.09)	**1e**−**6**	**1e**−**4**	**3e**−**3**
	*GON*	1 (0.3)	0.09 (0.04)			
20 db	*RKGW*	7.7 (4.6)	0.61 (0.25)	**9e**−**8**	**4e**−**6**	**2e**−**8**
	*GON*	2.5 (1.4)	0.2 (0.11)			
10 db	*RKGW*	23 (6.9)	1.28 (0.3)	0.06	0.18	**9e**−**12**
	*GON*	7.8 (4.3)	0.59 (0.29)			

For explanations, see the caption of Table 1

The *p*-values corresponding to the individual parameters are shown in the last three columns. Values shown in bold indicate a significant improvement achieved with the proposed method

**Table 6:** Comparison of RKGW and GON on the Biopathway data

*SNR*	Method	par${\scriptscriptstyle 10^{-2}}$	fun${\scriptscriptstyle 10^{-2}}$	$k_1$	$k_2$	$k_3$	$k_4$	$\frac{k_5}{k_6}$
40 db	*RKGW*	0.9 (0.1)	5.5 (0.7)	**5.8e**−**10**	**9e**−**6**	**1e**−**4**	**2e**−**6**	**1e**−**4**
	*GON*	1.9 (0.1)	5.9 (3.3)					
30 db	*RKGW*	1.3 (0.6)	6 (2.1)	**4.5e**−**3**	0.71	**8e**−**3**	**8e**−**6**	0.84
	*GON*	1.9 (0.2)	5 (2.5)					
20 db	*RKGW*	3.5 (2.5)	8.9 (5.4)	0.35	9e−3	**1.7e**−**6**	0.06	**3e**−**7**
	*GON*	2.1 (0.7)	9.5 (4.2)					
10 db	*RKGW*	5.3 (3.1)	16.6 (6.3)	**1e**−**6**	0.32	0.57	0.08	**0.01**
	*GON*	4.8 (3.1)	26.7 (19.6)					

For explanations, see the caption of Table 1

The *p*-values corresponding to the individual parameters are shown in the last five columns. Values shown in bold indicate a significant improvement achieved with the proposed method

**Table 7:** Comparison of RKGW and alternative methods on the Soft tissue mechanics data with 10 db SNR noise

Method	par	fun	*a*	*b*	*af*	*bf*
*RKGW*	0.95 (0.34)	0.13 (0.14)	**1e**−**3**	**3e**−**3**	0.24	**0.02**
*GON*	1.46 (0.81)	0.34 (0.21)				
*RKGW*	0.95 (0.34)	0.13 (0.14)	**1e**−**5**	**2e**−**4**	**7e**−**4**	**0.01**
$RKG_{rbf}$	1.12 (0.33)	0.18 (0.16)				
*RKGW*	0.95 (0.34)	0.13 (0.14)	**0.003**	**1 e**−**6**	**0.03**	**0.02**
$RKG_{mlp}$	2.27 (1.65)	0.37 (0.41)				

For explanations, see the caption of Table 1

The *p*-values corresponding to the individual parameters are shown in the last four columns. Values shown in bold indicate a significant improvement achieved with the proposed method

## B Warping example

Here we present a graphical example of the warping process for four DE models used in the simulation studies. First we show the true signal, noisy observations, initial interpolation and the improved interpolation using warping in (a). The warping function for different DE models is shown in (b). The true signal and interpolation in the warped domain are shown in (c). The gradients estimated using RKG and RKGW are plotted against the true gradients in the scatterplot in (d). The red trendline in (d) indicates when the estimated gradients equal the true gradients.

## C Comparison with Gaussian processes

We have included a comparison with a recently published alternative method based on Gaussian processes—the GPode model from Barber & Wang (2014), presented at ICML 2014. For this comparison, we used the authors’ own software. A naive application of this method, starting from a vague prior and no knowledge of the noise variance, consistently led to program crashes, related to singular covariance matrices and zero output probabilities. To avoid this problem, we first standardized all data to zero mean and unit variance, and transformed the differential equations accordingly by application of the chain rule of differential calculus. Let $\mu _i$ denote the mean of the *i*th state variable and $\sigma _i$ its standard deviation, then we define the standardized variable

26 $$\begin{aligned} {\tilde{x}}_i \; = \; \frac{x_i-\mu _i}{\sigma _i} \end{aligned}$$

By application of the chain rule we get

27 $$\begin{aligned} \frac{dx_i}{dt} \; = \; \frac{dx_i}{d{\tilde{x}}_i}\frac{d{\tilde{x}}_i}{dt} \; = \; \sigma _i \frac{d{\tilde{x}}_i}{dt} \end{aligned}$$

and hence

28 $$\begin{aligned} \frac{d{\tilde{x}}_i}{dt}\; = \; \frac{1}{\sigma _i} f({{\tilde{\mathbf{x}}}},{\varvec{\theta }}) \end{aligned}$$

This did not resolve the problem, though, as the program still consistently crashed with zero likelihoods, or, equivalently, minus infinite log likelihoods. To resolve this issue, we had to do three things: fix the noise variance to the unknown true value, make the parameter prior fairly informative (between zero and ten times the true value), and initialize the MCMC simulations with the unknown true parameter values. With the combination of these three fixes, the GPode method consistently avoided any singularity issues. However, the consequence is that the comparison with the method proposed in the present paper is no longer fair, as the GPode method uses substantial additional prior knowledge that would not be available in practice (and which our method is not given).

For a comparison of the results, we are facing the additional difficulty of having to compare a Bayesian with a frequentist method. For the Bayesian method (GPode), we show the posterior distribution of the parameters, obtained with MCMC. For the frequentist method (the proposed method), we show the distribution of the parameter estimates over 50 independent data instantiations. A comparison of these distributions is shown in Figs. 9, 10, 11 and 12. Apart from the Lotka–Volterra system, where the proposed method performs noticeably better than GPode, the results are essentially on a par, with sometimes the GPode and sometimes the proposed method performing slightly better. Note, though, that GPode achieved this level of performance only after the inclusion of substantial prior knowledge, whereas our method learned all the parameters from scratch.

## D Computational costs

**Table 8:** Comparison of computational costs

Model	CPU time
Lotka–Volterra	212*s*
FiztHugh–Nagumo	305*s*
Biopathway	158*s*
Soft tissue mechanics	71*s*

The computational costs for a single iteration of RKGW, using the data generated from the differential equations in Sect. 4. Computational costs with an explicit solution of the DEs were higher by a factor of 10–20

## E Detailed error distributions

The following figures show the distributions of the estimation errors for the various parameters, i.e. the differences between the estimated and the true parameters. From these distributions we obtained Fig. 3, which show the differences in the estimation errors between different methods (Figs. 17, 18, 19).

## Footnotes

1 The dependency on $\varvec{\varphi }_s$ is via $k_s$ (which has not been made explicit in the notation).

2 It would be more accurate to write $t_s$ and $\tilde{t}_s$ instead of *t* and $\tilde{t}$, which we avoid to reduce notational opacity.

3 The practical procedure is to increase $\lambda _t$ until the results are invariant wrt a further increase.

4 Recall that $t_i$ depends on *s*, so a more accurate (but cumbersome) notation would be $g_s(t_i) \rightarrow g_s(t_i^s)$.
